# An Evaluation of Clinical and Histopathological Aspects of Patients with Oral Submucous Fibrosis in the Background of Oral Squamous Cell Carcinoma

**DOI:** 10.1155/2018/4154165

**Published:** 2018-10-09

**Authors:** B. S. M. S. Siriwardena, K. L. T. D. Jayawardena, N. H. Senarath, W. M. Tilakaratne

**Affiliations:** ^1^Faculty of Dental Sciences, University of Peradeniya, Sri Lanka; ^2^Faculty of Allied Health Sciences, University of Peradeniya, Sri Lanka

## Abstract

**Background:**

The behavior and prognosis of oral squamous cell carcinoma (OSCC) is presumably different in patients with oral submucous fibrosis (OSF). The objective of this study was to assess the effects of demographic features, habits, and histopathological features in the transformation of OSF to OSCC.

**Methods:**

Data were extracted from the archives and histopathological evaluation and presence of nodal metastasis were recorded.

**Results:**

OSF was detected in 130 (48%) out of 273 OSCC patients. The mean age of presentation among OSF-positive patients was 57.7 years, while patients diagnosed only with OSCC had a comparatively higher age, 59.5 years. In the below 50 years of age group, presence of OSF with OSCC was less (40%). In the OSF-positive group, male to female ratio was 3.2:1. The common primary sites were buccal mucosa and tongue in both groups. Betel quid chewing was present in more than 95% of the sample. Betel chewing, smoking, and alcohol consumption were present in 26.15% of OSF-positive patients. Degree of fibrosis was neither associated with the level of histological differentiation of the tumor (p= 0.195) nor associated with the malignant transformation (p =0.373). Lymph node metastasis was not seen in 76.63% and 68.54% of the patients with and without OSF, respectively.

**Conclusions:**

High degree of prevalence of OSF was observed among the OSCC patients. There were also a male predilection and younger age at presentation in these patients. However, a significant association was not observed in the degree of fibrosis with malignant transformation or the level of histopathological differentiation of the tumor. Lymph node metastasis also failed to express a significant relationship with the presence of OSF.

## 1. Introduction

Oral squamous cell carcinoma (OSCC) is a critical health problem affecting millions of people worldwide. Even though the causes may vary regionally, the course of the disease and suffering prevails undeterred. The potentially malignant period of OSCC is an aspect that provides a beneficial approach to prevention. This period may be manifested as localized or generalized alterations of the epithelium, leading towards carcinogenesis.

Oral submucous fibrosis (OSF) is one such potentially malignant condition that subjects the oral cavity to a widespread alteration in morphology and physiology. The clinical manifestation comprises the classic triad: blanching of the mucosa, burning sensation on irritation with spicy food, and depapillation of the tongue. These will be followed by depigmentation of the lips and loss of elasticity of the mucosa with development of palpable fibrous bands in the oral cavity, progressing from the anterior region to the posterior region of the mouth. There are also apparent woody changes of soft palate and tongue, ultimately resulting in loss of mobility of the tongue along with restricted mouth opening [1–3].

This disease takes course due to the chronic exposure of the oral mucosa to carcinogens already identified in a constituent of the betel quid. In Sri Lanka, the betel quid is composed of betel leaf, areca nut, slaked lime, and fermented/dried tobacco leaf. The main etiological factor for OSF is identified as areca nut. Arecoline in areca nut is considered the principal agent while other alkaloids, polyphenols (tannins), and metallic ions (copper) are of contributory significance [4, 5]. This disease has a known malignant transformation rate varying from 7 to 13% [3].

The pathology takes effect due to the unusual proliferation of fibroblasts and increased production of collagen fibres extending from the immediate subepithelial region up to deeper muscle fibres. There are multiple molecular interactions in the extracellular matrix, leading to increased levels of Tissue Inhibitor of Matrix Metalloproteinases (TIMP-1, -2) [6, 7] and decreased level of Matrix Metalloproteinases (MMP-1). The number of inflammatory mediators and cytokines is also found responsible for the pathogenesis (e.g., TGF-*β*1 and *α*v*β*6) [8]. Furthermore, the epithelium may show atrophy and varying degrees of atypia while the lamina propria undergoes fibrotic changes, reduced vascularity, and inflammation [1–3].

Malignant transformation of OSF is described with possible genetic predisposition [9]. It may be further promoted in individuals with nutritional deficiencies and compromised immunity [4]. Carcinogenesis in OSF is also attributed to several biochemical mechanisms [4, 10–13]. The main contributors are described as arecoline-induced inactivation of tumor suppressor genes (P16) [13], damage to DNA (loss of heterozygosity and DNA double strand breakage) due to reactive oxygen species (ROS) [11, 14], epithelial and extracellular matrix interactions, and copper-induced angiogenesis along with abnormal collagen formation [4, 5]. Hypoxia, resulting from reduced vascularity, further promotes malignant transformation where hypoxia-induced factor –*α* (HIF-*α*) is known to be a key factor [4, 5, 10].

According to the existing knowledge, the prognostic indicators of OSCC with concomitant OSF have not been thoroughly investigated. The proposed unique nature of OSCC in this altered condition relates to younger age of presentation, better histological degree of differentiation of the tumor, and lesser potential for nodal metastasis [15]. However, there are opposing views supported by some studies where these relationships were not proven significant and also the countereffect had been observed [16].

The objective of the current study is to assess the presence of submucous fibrosis among biopsy-proven OSCC patients, followed up with histopathological evaluation of the degree of fibrosis, level of histological differentiation, and nodal metastasis. Similar studies have been carried out in India where a considerable number of patients present with this chronic progressive pathology, similar to Sri Lanka [17–19].

Nevertheless, there are no data of similar context in Sri Lanka regardless of the high numbers that are adding up to the patient population in each year. The current study aims to utilize patients' data where OSCC has already developed, to assess the prevalence of OSF in histopathological examination while excluding other pathologies that result in fibrosis, namely, scarring, keloid, gingival overgrowths, amyloidosis, oral lichen planus, anemia, and systemic sclerosis [20].

This study was carried out on similar grounds with the aim of evaluating the association of OSF with the nature of concomitant OSCC, in order to widen the knowledge beneficial to optimum management of these patients.

## 2. Materials and Methods

This is a retrospective analysis of patients diagnosed with OSCC. Data were obtained from the archives of the Department of Oral Pathology at the Faculty of Dental Sciences, Peradeniya. The sample comprised 273 patients.

Demographic and clinical information were recorded. Patients were categorized into two age groups, equal to or below 50 years and above 51.

Haematoxylin and eosin-stained sections of the OSCC specimens were obtained for histopathological analysis. These slides were reevaluated for presence of fibrosis, subepithelial hyalinization, and reduced vascularity as signs of OSF. The specimens with fibrosis were further subjected to grading of the severity of fibrosis. The OSCCs with OSF and the ones without were analyzed according to the degree of histological differentiation of the tumour. In addition to these data, the excisional biopsies were considered separately for the identification of lymph node metastasis, in both categories of OSCC patients.

Patients with biopsy-proven OSCC with adequate information were included in the study. The cases with recurrences or chemoradiation prior to surgical excision were excluded from the study.

## 3. Results

Among 273 OSCC patients, 130 (48%) patients were with features of OSF (Table 1).

### 3.1. Demographic Features

#### 3.1.1. Age

**Figure 1:**
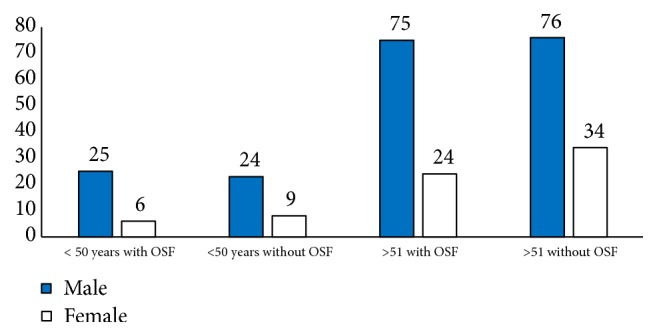
Distribution of OSCC with and without OSF among age groups.

The age distribution of the sample was analyzed. The patients were mostly above 50 years of age in both categories. However, the number of patients who were with OSCC at an age below 50 was greater in the OSF-positive group (23.8% vs. 21.9%). Similarly, the mean age of OSF + OSCC patients was 57.5 years while this value was 59.5 years for those without (Figure 1).

#### 3.1.2. Sex

The sample presented a male predilection, in both groups despite the presence or absence of OSF along with OSCC. The male to female ratio in OSCC only patients was 2.3:1. It was 3.2:1 in the group with OSF and OSCC. Similarly, the total study population showed a ratio of 2:7:1. This was not statistically significant at p<0.05.

#### 3.1.3. Oral Habits

**Figure 2:**
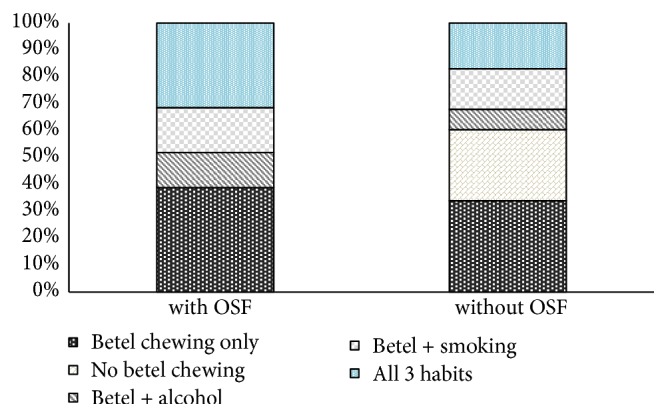
Distribution of habits and its relationship with OSF.

Information regarding the habits was obtained with regard to betel quid, smoking, and alcohol consumption. Most of the patients had positive habit history. Betel quid chewing with or without smoking or alcohol consumption was observed in all OSF-positive patients. This was statistically significant at p< 0.05 (p= 0.00022). All 3 habits were seen in 26 patients where 65.38% of them were with OSF. In addition, 39 patients were only having the habit of betel quid chewing where 53.84% of them were with OSF. Some patients [6] without OSF in OSCC were with no known risk habits. None of the patients with OSF were devoid of a habit history (Figure 2).

#### 3.1.4. Site of the OSCC

**Figure 3:**
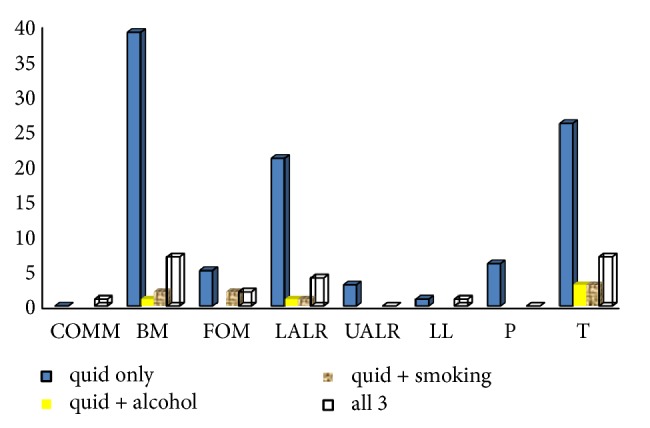
Relationship with habits including betel quid and primary site.

The commonest site among these patients was buccal mucosa followed by tongue. This was comparable to both groups despite the presence or absence of OSF. In OSF patients 34.35% were with OSCC in buccal mucosa while 29% were in tongue. The habit associating the relationship with the site of OSCC in OSF-positive patients can be demonstrated as below (Figure 3).

### 3.2. Histopathological Features

#### 3.2.1. Degree of Fibrosis and Degree of Histological Differentiation of the Tumour

The majority of the OSCCs were well differentiated followed by moderately and poorly differentiated OSCCs. The degree of differentiation was observed in both groups, namely, with or without OSF. It was revealed that the majority of the OSCCs from both categories are mainly well differentiated. It was 54.9% in the former and 64% in the latter. Presence or absence of OSF had not made a significant difference to the differentiation of the tumor (p=0.373).

**Figure 4:**
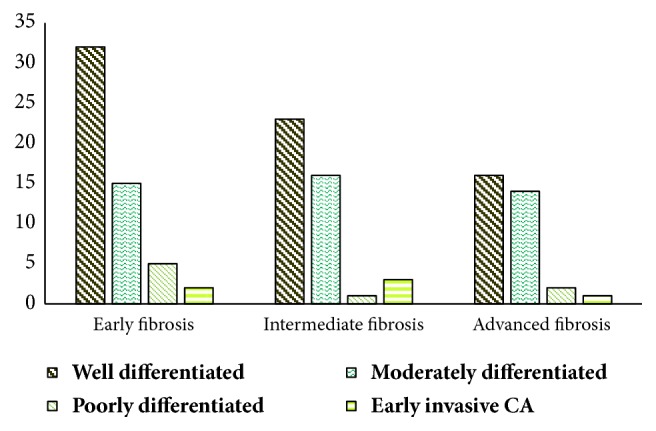


Most of the OSF patients were having early fibrosis (41.9%). Intermediate and advanced fibrosis were seen in 32.8% and 25.1%, respectively (Figure 4). The distribution of OSCC among different grades of fibrosis (early/intermediate/late) was also assessed. In each category, the highest number was seen as well-differentiated OSCCs. There was no significant relationship between degree of fibrosis and histological differentiation (p=0.195).

#### 3.2.2. Metastasis of the Tumor

The total number of OSCC excisions with cervical neck dissections was 200. Among these 106 patients were with OSF while 96 were only with OSCC. Lymph node metastasis was seen more among OSCC only patients (30.8% vs 22.6%). But this association was not statistically significant (p=1.89) (Table 2). The degree of fibrosis was assessed in relation to metastasis. In the analysis, 5 (17.85%) cases with advanced fibrosis showed positive lymph nodes while the values for intermediate and early fibrosis were 29.4% and 20.9%, respectively.

## 4. Discussion

The management guidelines of OSCC mostly follow curative intent unless the tumor stage or the patient's condition demands otherwise. Yet poor prognosis of OSCC patients is commonly observed. This draws attention to deficits in intervention.

When different OPMDs are assessed, it is acceptable that OSCC may depict features characteristic to the nature of the initial lesion. Leukoplakia, erythroleukoplakia, proliferative verrucous leukoplakia, verrucous hyperplasia, oral lichen planus, and OSF are such OPMDs where the transformation may occur into OSCC. However the clinical signs and symptoms of the lesions vary from one to another.

Thus, the possible unique nature of OSCC in OSF has been assessed based on the hypothetical phenomenon that it may show a difference in prognosis.

In previous studies it has been shown that the submucosa, in OSF, undergoes pathological changes due to excessive fibrosis, abnormal collagen synthesis, reduced vascularity, and hypoxia [1–3, 6–8]. In turn, their pathway of malignant transformation takes course under the influence of genetic and molecular alterations [4, 5]. The basis for expecting early detection, less invasion, and metastasis may be due to excessive collagen fibre production with increased crosslinkages that are not degraded by collagenase. However, the reduced vascularity in an environment with fibrosis prolongs the accumulation of carcinogens that permeate the mucosa and enables their action to last longer [4, 5]. Ultimately when tumorigenesis takes place, they may possess different prognostic attributes in comparison to OSCC in an environment that lacks OSF. Presence of OSF was observed in 48% of the patients in the current study. This lies within the range 25.77% [18] to 66% [21], observed in the literature.

The 5-year survival rates of patients remain 70-80% with Stage 1 and 2 OSCCs while they drastically reduce to 40% in latter stages of the disease [22, 23]. Thus early detection is an important prerequisite for better prognosis of OSCC patients which relates favourably to OSF patients. Another outcome of delayed presentation is regional metastasis. Regional metastasis is reported among 34-50% [24] of the patients at the time of diagnosis due to the asymptomatic nature of malignant lesions. The expected early presentation of OSF patients is attributed to the functional limitations experienced by these individuals. In the contrary, those patients who have OSCC without concomitant OSF do not normally experience pain and discomfort till the lesion progresses to a functionally limiting stage. Hence, the majority of patients from the OSCC only group belong to 5-7th decades of life.

This is comparable to the current study as well. When the patients were categorized as below and above 50 years of age groups, the majority were above 50 in general (76.55%). However, as a percentage in OSF with OSCC patients, the patients below 50 years were more (23.8% vs. 21%). A separate analysis was carried out where the categorization was below and above 55 years of age. In this analysis 39.69% of the patients with OSF were below 55years whilst it was 33.09% for the OSCC without OSF group. Yet, there was no statistically significant relationship. According to the literature, mean age values for OSF with OSCC patients range from 44.54 [19] years to 45.8 [16] years. On the contrary, patients without OSF have mean age values in the range of 47.78 years [19] to 55.9 years [16]. Sarode et al. [25] and Hashmi et al. [19] have concluded that OSCC patients with OSF present at a significantly younger age than those without. The current study has age values lying above the ranges presented in previous researches. Mean age of OSF with OSCC was 57.7 years and it was 59.5 years for the OSCC patients without OSF. We presume that the difference may attribute to delay in presentation, delay in onset of habits, and even a difference in the type of habits. Therefore, a detailed epidemiological analysis is required in order to clarify the above differences.

Male predilection is observed in the literature when it comes to most OPMD conditions and OSCCs. This is owing to the risk habits which are being practiced more commonly among males, relative to the female community. This may also account for multiple simultaneous habits such as smoking and alcohol, which have synergistic effects on carcinogenesis [26]. The current study is supportive of this finding. The sample was mainly comprised of males (73%) and it was common to both categories of patients. Some studies present staggeringly high number of male patients when considering patients with OSCC in OSF. Chaturvedi et al. [17] state a male to female ratio of 10:1 in OSF-positive group, while it was only 3.2:1 for OSCC patients. Gue et al. [16] have found this ratio to be 32.3:1 and 2.3:1 ratios in OSF-positive and -negative categories, respectively. In addition, Sarode et al. [25] state that male predilection in patients having OSF with OSCC is statistically significant (p<0.007). However, we were unable to identify such a significant association with gender in the current study. The male:female ratio of patients with OSCC in OSF was 3.1:1 whilst it was 2.3:1 for OSCC patients without OSF. This ratio in the total sample was 2.7:1, thus giving comparable results to existing literature, with an apparent male predilection.

The habit history is an important aspect of OSCC patients. It is applicable to prognosis and to treatment. The rationale of defining the behavior and outcome of OSCC in OSF is also closely linked to its aetiopathogenesis. Therefore, an analysis of habit history is undoubtedly required. In the current study, betel quid consumption was observed in all the patients with OSF and it was a statistically significant finding. Similarly, Singh et al. [21] have reported positive chewing habit in 96%, in his study on nodal status of OSF patients with OSCC. In the current study, all three habits were seen in 26 patients where the majority were positive for OSF (65%). This is a possible effect of mutagenic substances in the altered connective tissue environment that may result in a more vulnerable epithelium [4, 5].

Six patients were observed to have no known risk habits even though they were positive for OSCC which requires further investigation.

The site of OSCC is believed to be a direct indication of the type and nature of oral habits. Thus the Sri Lankan population is known to present with OSCC more commonly in the buccal mucosa, tongue, or the floor of the mouth rather than other subsites of the oral mucosa [27]. The practice of placing quid in the buccal sulcus while chewing and moving it into different regions of the mouth with the use of the tongue is observed in habitual betel quid chewers. This practice is different from the individuals who use smokeless tobacco or commercially prepared areca products that are placed in contact with a specific region of the oral cavity for absorption. It is also different from the practice of smoking tobacco. While attesting the causal relationship of the aetiologic factors it enhances the predictability of OSCC development in particular sites of the oral cavity. Similar association was observed in this study where OSCC was common in the buccal mucosa, in the tongue and thirdly in the lower alveolar ridge. Presence or absence of OSF did not have an apparent effect on the site of the OSCC. Several studies present comparable findings with regard to the site of OSCC [17, 27]. A research in India has further revealed a significantly higher occurrence of OSCC in the buccal mucosa of OSF patients when compared to the counterparts of their study [19]. Yet, a similar significance was not apparent in this study maybe as a result of the sample size.

Previous studies demonstrated a significant relationship between thicknesses of fibrosis and the grade of dysplasia in OSF patients [28]. It is presumed that degree of dysplasia may act as the transition stage between the normal epithelium and the OSCC, depicting a higher risk of malignant transformation in time, unless intervened. Therefore, it could be expected that larger number of OSCCs would occur in a background of advanced fibrosis which promoted dysplastic changes in the epithelium, more promptly. However, our assessment of fibrosis in OSCC patients did not demonstrate a statistical significance so as to predict that a higher number of OSCCs occur in patients with advanced fibrosis rather than those who are in the early stage. Similar findings have been made in a previous study where a significant association was not observed between degree of fibrosis and dysplasia or malignant transformation [12]. Thus, there is an avenue for further studies to evaluate fibrosis associated with prognosis of OSF patients while modifying the treatment modalities to better suit our main objective of eliminating the risk of malignancy rather than fibrosis alone.

When the histopathological features were assessed, it was observed in the literature that OSCC in OSF shows favourable histopathological features.

Better degree of histological differentiation of the tumor was one such feature. Chaturvedi et al. [17] have observed that OSCC without OSF patients had significantly higher number of poorly differentiated OSCCs than in the ones with OSF (13.5% vs. 13.4%). Gadbail et al. [15] have also revealed significantly high numbers of well-differentiated OSCCs in patients with OSF. A rationale which justifies the effect of fibrosis on the level of differentiation is yet to be proven scientifically. In this study, both groups had a large number of well-differentiated tumours. Hence, the current study does not provide supportive evidence to the previous findings in several researches.

Nodal metastasis is another feature which is presumed to be less in OSF patients due to the obstruction created by fibrosis in the lamina propria. Gadbail [15] and Chaturvedi et al. [17] have observed results which are supportive of this feature. Singh et al. [21] have further stated that nodal metastasis is significantly less in T4 stage patients with OSF where the percentages were 28.6% versus 81.1% in OSCC without OSF. On the contrary Guo et al. [16] report more metastasis and recurrence in OSF patients with OSCC. The current study showed that nodal metastasis was less in patients with OSF compared to the ones without (22.6% vs. 30.8%). Yet, this finding did not approach statistical significance.

Other features pertaining to the prognosis of OSCC patients were observed in multiple studies where they indicated less perineural invasion and less lymphovascular invasion [17, 19]. There was an exception in one feature which relates to poor prognosis, in a single study, where the extracapsular spread was more in OSF patients with OSCC (8% vs. 6%) [19]. There was also evidence of less tumor thickness among OSF-positive patients, which may be an outcome of the impenetrable nature of the corium in these patients. Therefore, it could be accepted that OSCC in OSF possesses better prognosticators in comparison.

The current study was carried out in subjects with histopathologically proven OSCCs where several findings were supportive of the already suggested unique characteristics of concomitant OSCC in OSF. Nevertheless, it is necessary to carry out a prospective analysis of a larger sample size, for better clarification and justification of the findings in the literature.

## 5. Conclusion

Concomitant OSCC in OSF is common among males and maybe among younger patients. These tumors show well-differentiated histology and less lymph node involvement. However, a statistical significance was not observed among these variables, when comparing the OSCC patients with OSF to those who are without.

It can also be suggested that the degree of fibrosis may not be a determinant of malignant transformation, higher level of differentiation, or less nodal metastasis.

## Figures and Tables

**Table 1:** Summary of the results.

	**Patients with OSF+OSCC**	**Patients with OSCC**	**P value**
**Age **	31 patients <50 years	33 patients < 50 years	
	99 patients > 51 years	110 patients >51 years	
**Sex**	M: F	M:F	
	3.2:1	2.3:1	
**Habits**			
Betel quid chewing	99	23	<0.05*∗*
Quid with alcohol or smoking	13	10	
Alcohol and smoking	0	5	
All 3 habits	22	9	
**Primary site**			
Commissure	1	7	
Buccal mucosa	47	51	
Floor of the mouth	9	10	
Tongue	39	38	
Alveolar ridge (upper and lower)	30	33	
Lip	2	1	
Palate	0	5	
**Degree of fibrosis and Histological differentiation **			
Early fibrosis:			
EISCC	02	02	P=0.195
WDSCC	32	91	
MDSCC	15	42	
PDSCC	05	06	
Intermediate fibrosis:			
EISCC	03		
WDSCC	23		
MDSCC	16		
PDSCC	01		
Advanced fibrosis:			
EISCC	01		
WDSCC	16		
MDSCC	14		
PDSCC	02		
**Lymph node metastasis **	24 (22.6%)	29 (30.8%)	P=1.89

*∗*Significant association between betel quid and OSF P<0.05, EISCC- early invasive SCC, WDSCC-well DSCC, MDSCC- moderately DSCC, PDSCC- poorly DSCC (DSCC-differentiated squamous cell carcinoma).

**Table 2:** Nodal metastasis and its relationship with OSF.

	Metastasis present	Metastasis absent	Total
OSF + OSCC	24 (22.6%)	82 (77.3%)	106
OSCC only	29 (30.8%)	65 (69.1%)	94

## Data Availability

The data used to support the findings of this study are available from the corresponding author upon request.
